# Effect of total body irradiation lung block parameters on lung doses using three‐dimensional dosimetry

**DOI:** 10.1002/acm2.13513

**Published:** 2022-01-05

**Authors:** Samuel M. H. Luk, Kent Wallner, Mallory C. Glenn, Ralph Ermoian, Mark H. Phillips, Yolanda D. Tseng, Minsun Kim

**Affiliations:** ^1^ Department of Radiation Oncology University of Washington Seattle, Washington USA; ^2^ Department of Radiation Oncology University of Vermont Medical Center Burlington Vermont USA; ^3^ Clinical Research Division Fred Hutchinson cancer Research Center Seattle, Washington USA

**Keywords:** lung dose, radiation oncology, total body radiation

## Abstract

**Purpose:**

Total body irradiation (TBI) is an integral part of stem cell transplant. However, patients are at risk of treatment‐related toxicities, including radiation pneumonitis. While lung dose is one of the most crucial aspects of TBI dosimetry, currently available data are based on point doses. As volumetric dose distribution could be substantially altered by lung block parameters, we used 3D dosimetry in our treatment planning system to estimate volumetric lung dose and measure the impact of various lung block designs.

**Materials and methods:**

We commissioned a TBI beam model in RayStation that matches the measured tissue‐phantom ratio under our clinical TBI setup. Cerrobend blocks were automatically generated in RayStation on thoracic Computed Tomography (CT) scans from three anonymized patients using the lung, clavicle, spine, and diaphragmatic contours. The margin for block edge was varied to 0, 1, or 2 cm from the superior, lateral, and inferior thoracic borders, with a uniform margin 2.5 cm lateral to the vertebral bodies. The lung dose was calculated and compared with a prescription dose of 1200 cGy in six fractions (three with blocks and three without).

**Result:**

The point dose at midplane under the block and the average lung dose are at the range of 73%–76% and 80%–88% of prescription dose respectively regardless of the block margins. In contrast, the percent lung volume receiving 10 Gy increased by nearly two‐fold, from 31% to 60% over the margins from 0 to 2 cm.

**Conclusions:**

The TPS‐derived 3D lung dose is substantially different from the nominal dose assumed with HVL lung blocks. Point doses under the block are insufficient to accurately gauge the relationship between dose and pneumonitis, and TBI dosimetry could be highly variable between patients and institutions as more descriptive parameters are not included in protocols. Much progress remains to be made to optimize and standardize technical aspects of TBI, and better dosimetry could provide more precise dosimetric predictors for pneumonitis risk.

## 1 INTRODUCTION

Total body irradiation (TBI) is an integral part of stem cell transplants (SCT), used for treatment of leukemia, lymphoma, and several benign conditions.^[^ ^1^ ^]^ SCTs can be life‐saving procedures. However, patients are at substantial risk of treatment‐related, life‐threatening toxicity, including veno‐occlusive disease (VOD), chronic graft‐versus‐host disease (cGVHD), and radiation pneumonitis (RP).^[^ ^2^ ^]^

While external beam radiation therapy has evolved to remarkable 3D and 4D sophistication, technical aspects of classical TBI such as treatment planning and dosimetry remain relatively crude, typically delivered with minimally modified broad beams and 1D dosimetry. Beam modifications are limited to spoilers, head‐to‐toe compensators, and partial lung transmission blocks.^[^ ^1^, ^3^ ^]^ The latter are the most important, but least precise of the modifications.

Lung dose is one of the most dose‐limiting aspects of TBI dosimetry. Multiple studies have shown steep normal tissue dose–response curves, with the likelihood of RP rising sharply between a lung dose of 5–12 Gy.^[^ ^4^, ^5^, ^6^ ^]^

**FIGURE 1:**
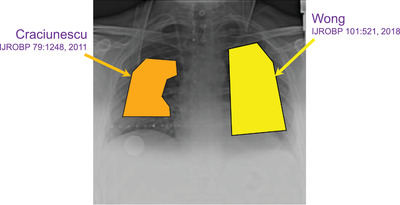
Right vs. left lung blocks drawn according to two authoritative publications

Given the steep RP dose–response over a relatively narrow range, authoritative publications provide detailed descriptions for drawing lung blocks.^[^ ^1^, ^7^ ^]^ Guidelines are intended to standardize practice and limit lung dose to lower the risk of RP. Standardized block parameters are desirable, but there can be considerable variation in defining and implementing the recommendations. Figure 1 illustrates two lung blocks drawn according to guidelines from two mainstream publications. Areas of inconsistency include margins from block to chest wall/diaphragm/spine, flat versus curved inferior borders, inclusion of the lung apex, number of half‐value‐layer (HVL), and whether to block the cardiac silhouette. Block parameter variability could substantially alter lung dose parameters and the likelihood of RP.

The published lung dose parameters are simply point doses monitored at the skin, under the lung block. Point dose specifications may have limited relation to the complex volumetric dose distributions resulting from patient anatomy, transmission block thickness, in‐patient scatter, and variable block margins. While point doses have correlated with the likelihood of RP, it is likely that more accurate dosimetry would enhance the ability to predict and prevent the pulmonary toxicity. Previous investigators have explored 3D TBI dosimetry.^[^ ^8^, ^9^, ^10^, ^11^ ^]^ The work presented here builds on those efforts, with the ultimate goal of optimizing lung block parameters for individual patients.

Spurred by concern over vague and inconsistent lung block criteria and 1D point measurements, we commissioned a TBI beam model in our treatment planning system (TPS) and calculated 3D dosimetry at extended distance with lung blocks. The updated TPS was used to determine more detailed, volumetric lung dose resulting from various lung block designs.

## 2 METHODS

### 2.1 TBI set‐up and current practice

The set‐up parameters conform to standard recommendations.^[^ ^1^ ^]^ Patients are treated anterior–posterior and posterior–anterior (AP/PA), standing or decubitus using a linear accelerator (Elekta Synergy). The patient's midline is located at 475 cm from the 18 MV photon source. A 1.2 cm Lucite beam spoiler is placed 40 cm away from the patient's midline to increase the surface dose. Patient‐specific lead compensators are placed on the block tray in the gantry head to achieve a uniform dose to the monitoring points.

In current clinical practice, treatment monitor units (MU) for TBI patients are computed to deliver the prescription dose to the patient's midline at the umbilicus level using a correction factor‐based spreadsheet that is currently used in our practice accounting for tissue maximum ratios (TMR), spoiler factor, tray factor, off‐axis ratios, and relative output factor. Dose uniformity at the dose monitoring points, that is, head, neck, upper and lower mediastinum, umbilicus, knee, and ankle, is calculated in the same manner. The thickness of patient‐specific lead compensators is adjusted to ensure that the dose uniformity is within 10% from the prescription dose.

### 2.2 Commissioning and validation of TBI beam model in the treatment planning system

**FIGURE 2:**
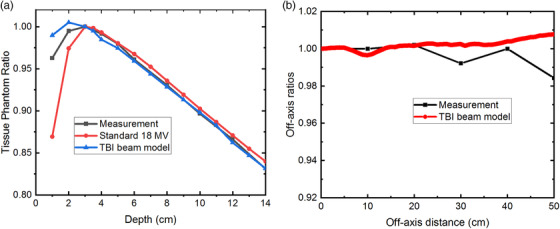
(a) Comparison of standard and total body irradiation (TBI) model in treatment planning system (TPS), vs. measured tissue phantom ratios (TPR) values. (b) Comparison of off‐axis ratio of TBI model in TPS vs. measured values

The standard 18 MV beam, previously commissioned at 100 source‐to‐axis distance (SAD), was copied into the RayStation TPS (Version 8B, RaySearch Laboratories, Stockholm, Sweden). RayStation uses collapsed cone algorithms on dose calculation. Tissue phantom ratios (TPR) with a reference depth of 3 cm under the TBI setup were computed in the TPS and compared with TPR measured under the TBI setup. TPS‐computed TPRs differed from the measured values by 12% in the buildup region and 3% beyond 3 cm. We modified the energy spectrum of the standard 18 MV beam model by increasing the low energy components. The electron fluence was adjusted to best fit the TPS‐computed TPR with the measured TPR in the buildup region. The final beam model allows the computed TPR values to agree with the measured values to within the error of up to 2% at 1 cm depth and 0.4% beyond a depth of 3 cm in a large square water phantom (Figure 2c).

A water‐equivalent bolus structure was added to the patient surface to simulate the patient‐specific lead compensators used at the treatment head to achieve the required dose uniformity (i.e., less than 10% variation from the prescription dose) in the patient accounting for a different thickness at a different part of the body. The profile of the TBI beam model with this bolus is flat, agreeing with measured values to within 1% up to 40 cm from center (Figure 2). The measured data are less accurate at larger off‐axis distances than 40 cm, due to limited solid water scatter.

**FIGURE 3:**
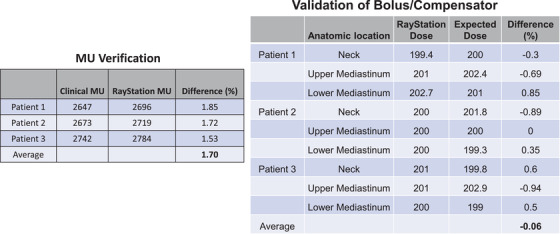
Monitor unit (MU) verification and validation of bolus/compensator on dose uniformity by comparing the treatment planning system (TPS) with the clinically calculated MU and dose distributions

The beam model was validated by comparing the treatment MU obtained from the spreadsheet used in current clinical practice with the MU computed in the TPS using the newly commissioned TBI beam model. The average difference in MU of three anonymized patients was 1.7% (Figure 3). The effect of water‐equivalent bolus structure on mimicking patient‐specific lead compensators was validated by comparing the calculated off‐axis dose at neck, upper mediastinum, and lower mediastinum using the current clinical practice spreadsheet versus TPS. The uniformity achieved in RayStation is within 1% at all dose monitoring points confirming that our method in TPS is consistent with what we do in current practice. Moreover, we have point doses measured at different points (including head, lung, umbilicus, knee, and ankle) on all patients using diodes as part of the clinical protocol, and the measured doses agree within 10% of the prescription dose with patient‐specific lead compensators. This also agrees with the TPS computed values using the commissioned TBI beam and the water‐equivalent bolus structures validating our method.

### 2.3 Lung blocks in treatment planning system

**FIGURE 4:**
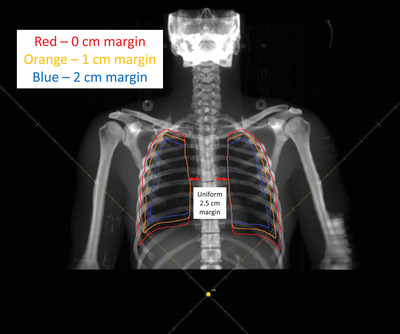
Illustration of lung blocks drawn with 0, 1, and 2 cm block‐to‐chest wall, clavicles, and diaphragm margins

**FIGURE 5:**
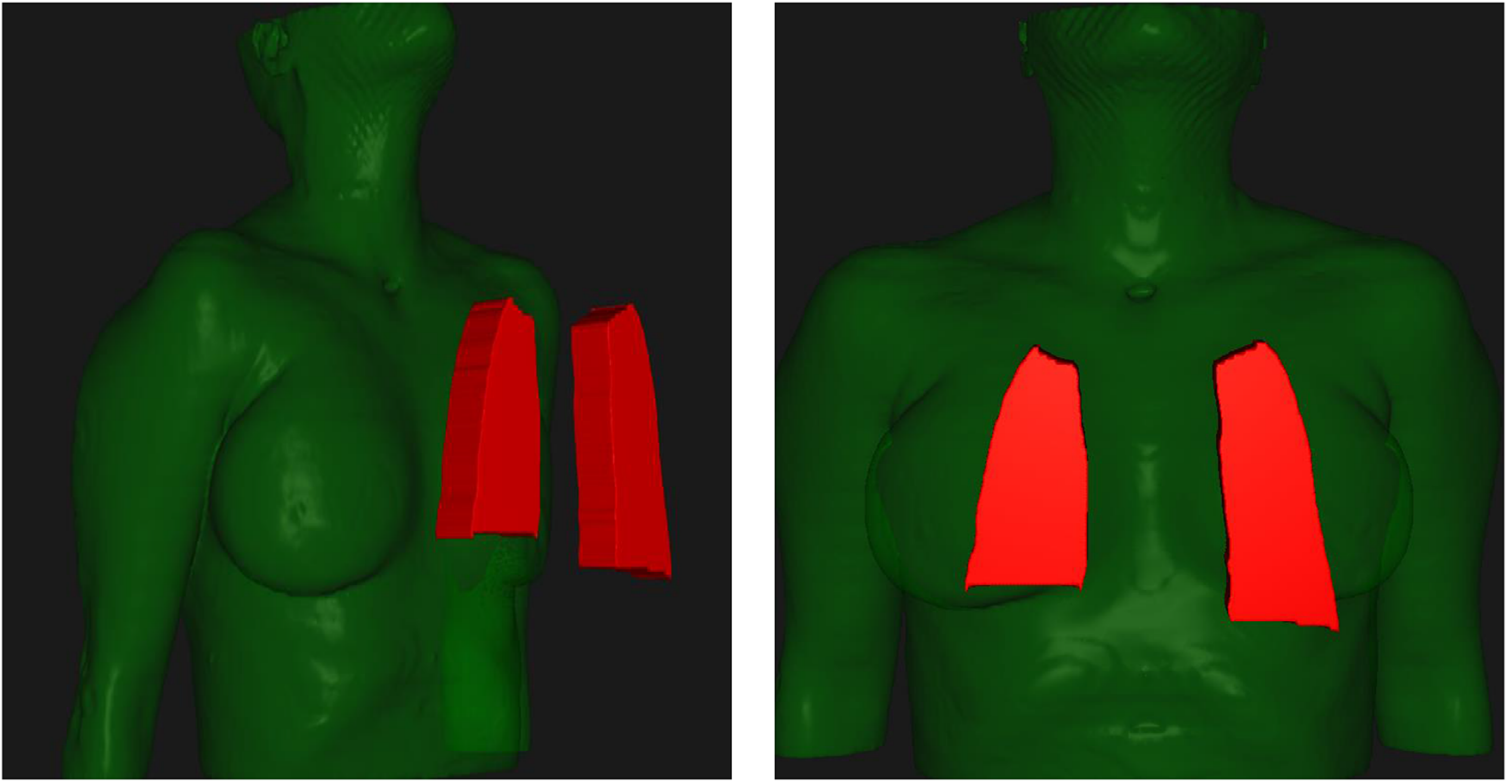
Illustration of the three‐dimensional (3D) positions of anterior–posterior (AP) lung blocks in the treatment planning system (TPS). The water‐equivalent bolus and beam spoiler are not shown in the figure

Using the18 MV TBI beam model, a script was created to automatically generate the TBI setup and lung blocks on thoracic CT scans from anonymized patients. Lung blocks were generated using the lung, clavicle, spine, and diaphragmatic contours and the margin for the block edge was varied to 0.0, 1.0, or 2.0 cm from the superior, lateral, and inferior thoracic borders (Figure 4). A uniform 2.5 cm margin lateral to the vertebral bodies was used. The generated 4.4 cm, or 2 HVL Cerrobend blocks are considered support structures in the TPS, located 25 cm away from the patient midplane (reflect current practice of patients treating in the standing position). The 3D positions of the lung blocks in the TPS are shown in Figure 5 (only AP blocks are shown).

### 2.4 Dosimetric evaluation

Lung dose was calculated using 0.0, 1.0, and 2.0 cm block margins, with a prescription dose of 200 cGy per fraction for 6 fractions, delivering 1200 cGy total to unblocked tissue. The calculation was computed in the TPS with a dose grid that covers the volume between the anterior and posterior lung blocks. The dimension of the dose grid is roughly 60 cm × 200 cm × 80 cm (right–left, inferior–superior, PA) with a 0.5 cm resolution, resulting in around six million voxels. Three fractions are delivered with two HVL lung blocks and three fractions without. This results in a nominal dose of 62.5% of the prescription dose to lungs. Here, we consider the ‘‘nominal” dose as the expected point dose in the lung under the block assumed with HVL, for example, the expected dose from two HVL lung blocks is 25% of the prescription dose. The 200 cGy × 6 fractions prescription scheme is used as it is the most commonly used TBI regime at major centers,^[^ ^12^ ^]^ but other TBI treatment regimens would also benefit from understanding the relationship between lung block margins and lung dose.

## 3 RESULTS

**FIGURE 6:**
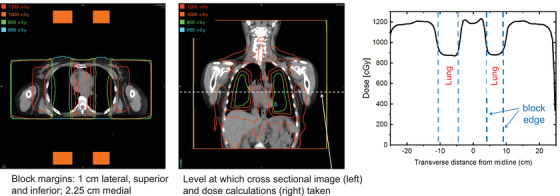
Transverse and coronal isodose lines and dose profile for one patient

The TPS calculations were substantially different than expected from the two HVL lung blocks. The point doses at midline under the block center stayed within the range 73%– 76%, regardless of block margins, higher than the expected (nominal) 62.5% of prescription, presumably due to the combination of additional scattering from the extended distance, the blocks themselves, and within‐patient scatter. The dose profile is highly variable across the thorax (Figure 6). The dose under the block increases rapidly off‐center, such that much of the tissue under the block receives close to the full prescription dose, rather than the nominal dose.

**FIGURE 7:**
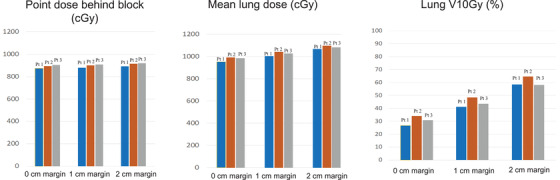
Point doses under the block center, average lung dose, and V10Gy dose with 0, 1, and 2 cm margins

The average central point dose did not change substantially as the block margins expanded from 0.0 to 1.0 to 2.0 cm, increasing from 885±11 cGy to 891±12 cGy to 902±11 cGy, respectively (Figure 7). The average lung dose increased from 977±21 cGy to 1025±19 cGy to 1043±15 cGy (80%– 88% of the prescription dose) with margins increasing from 0.0 to 1.0 to 2.0 cm. The average lung dose of unblocked fraction is 216 ± 3 cGy, which is 8% higher than the single‐fraction prescription (2 Gy). The average lung dose of blocked fractions with margins increasing from 0.0 to 1.0 to 2.0 cm is 110±5 cGy, 126±6 cGy, and 145±5 cGy, respectively (55%– 72.5% of the single‐fraction prescription dose).

In contrast, the percent lung volume receiving 10 Gy (V10Gy) increased by nearly two‐fold, from 31%±4% to 44%±4% to 60%±4% over the same block margin range. Overall, the central under‐block point dose, average dose, and V10Gy increased by approximately 3%, 8%, and 100%, respectively.

## 4 DISCUSSION

The adoption of lung shielding has led to a decrease in RP.^[^ ^1^ ^]^ However, the risk still remains in the range of 10%–20%. Inaccurate lung dosimetry may contribute to the still‐substantial RP risk. To our knowledge, the calculations reported here are the first calculations with a modern TPS, to more accurately quantify the 3D dose–volume metrics delivered with typical clinical methodology using AP/PA beam arrangement at an extended SAD. The TPS‐derived 3D dose is substantially different than expected by the nominal dose assumed with HVL lung blocks, due to increased scattering of photons in air at an extended distance, beam spoiler and compensator, and inter‐patient scattering, all of which increase low energy photons in the energy spectrum.

More descriptive parameters, including average lung dose, V10Gy, the measured dose under the block, and the average dose to lung when it is not shielded are not specified in protocols and leave room for highly variable dosimetry between patients and between institutions.^[^ ^12^ ^]^ Given the high radiation sensitivity of lung tissue and steep dose–response, we believe that the use of point doses is insufficient to accurately gauge the relationship between dose and what would otherwise be considered the unpredictability of RP. It is likely that more accurate, TPS‐based lung dosimetry could lead to more precise control of lung parameters, and ultimately, to a lower risk of RP.

Just how much help more detailed dose determinations would be in predicting and preventing RP depends on which lung parameters are most closely associated with toxicity. To our knowledge, most published clinical protocols are limited to using point‐dose measurements, such that the relationship between the 3D dosimetry and clinical outcomes is largely unknown. If the average lung dose is the most important, inconsistent block edge methodology would not matter much, since the average lung dose is relatively insensitive to block margins. However, if higher dose parameters (V10Gy or V12Gy) are important, the risk of pneumonitis could be substantially altered by differences in block parameters. The use of more descriptive parameters might help explain the unpredictable and persistent risk of RP that exists despite use of current guidelines.^[^ ^13^ ^]^ We believe that future studies, using 3D dosimetry, might allow better evaluation and prevention of pneumonitis risk.

The another aspect of variable lung block margins is alteration of dose to non‐lung regions. Larger lung blocks would decrease the lung dose but may deliver a lower dose to thoracic bone marrow, which could decrease the anti‐leukemic effect. In our study, we did not vary the margin in the medial direction toward vertebrae but fixed it to a 0 cm margin. Quantifying the potential detriment in the clinical effectiveness of larger lung blocks is beyond the scope of this paper. However, it is likely that 3D lung dosimetry would provide more information to determine the dose–response relationships between lung dose, pneumonitis, and cure rates.

Other investigators are working to improve the quantification and delivery of TBI lung dose through different techniques than a 2D approach. Wong and others have adopted Volumetric Modulated Arc Therapy (VMAT) delivery, allowing for sophisticated calculation and control of lung dose.^[^ ^1^ ^]^ While there is excitement about VMAT for TBI, there is also concern regarding the inhomogeneous dose rates and geographically heterogeneous delivery with multiple isocenters. VMAT‐like delivery may someday be standard. In the meantime, we believe that the dosimetric methods described here could be used to better guide the design and implementation of lung blocks in standard AP/PA positioning, to allow more precise control over lung dosimetry.

## 5 CONCLUSION

Much progress remains to be made to optimize and standardize technical aspects of TBI. The work presented here is a step toward improving TBI dosimetry. It should eventually lead to more accurate dose determinations that should lead to better informed treatment planning, delivery, and ultimately, better control of RP risk. Ideally, future clinical studies will incorporate more detailed and standardized, optimal lung blocking.

## FUNDING

None.

## CONFLICT OF INTEREST

The authors declare that there is no conflict of interest that could be perceived as prejudicing the impartiality of the research reported.

## AUTHOR CONTRIBUTIONS

Samuel M. H. Luk, Kent Wallner and Minsun Kim conceived the presented idea, and wrote the manuscript with the support from Ralph Ermoian and Yolanda D. Tseng. Samuel M. H. Luk and Mark H. Phillips carried out the commissioning measurement of TBI beam and developed the beam model in TPS. Samuel M. H. Luk and Mallory C. Glenn created the patient model, collected, and analyzed the data in TPS. Kent Wallner, Ralph Ermoian, and Yolanda D. Tseng contributed to the medical aspect of the TBI.

## Data Availability

Research data are stored in an institutional repository and will be shared upon request to the corresponding author.
